# Supplementary material for: Global coral genomic vulnerability explains recent reef losses
Source: Nat Commun. 2025 Dec 19;17:896. doi: 10.1038/s41467-025-67616-5 (PMC12830885; doi:10.1038/s41467-025-67616-5)
Supplement: Supplementary file 1 — Supplementary Information [file 41467_2025_67616_MOESM1_ESM.pdf]

Supplementary Materials for

Global coral genomic vulnerability explains recent reef losses

Oliver Selmoni, Phillip A. Cleves, Moises Exposito-Alonso

Correspondence to: oliver.selmoni@gmail.com, pacleves@berkeley.edu, moisesexpositoalonso@gmail.com

This PDF file includes:

Tables S1 to S5
Figs. S1 to S22

Table of content

Supplementary Tables **3**
Table S1. Genomic datasets. 3
Table S2. Coefficients of models explaining genetic distances between samples. 4
Table S3. Gene ontology enrichment analysis. 5
Table S4. Coefficients of Acropora cover change models. 6
Table S5. Parameters of Mutations-Area Relationship Linear Mixed Model. 7
Supplementary Figures **8**
Figure S1. Workflow of the study. 8
Figure S2. Sequencing read mapping stats. 9
Figure S3. Distribution of SNPs across genomes. 10
Figure S4. Principal component analysis of sequencing and alignment statistics. 11
Figure S5. Principal component analysis of genotype matrices. 12
Figure S6. Shared SNPs and % of non-identical genotypes across datasets. 13
Figure S7. Genetic proximity of all samples from the Acropora datasets. 14
Figure S8. Heat stress variables distribution across datasets. 15
Figure S9. Missing values, allele frequencies statistics and allele frequency correlation 16
Figure S10. Cross-entropy estimation of the number of ancestral populations. 17
Figure S11. Validation of overlapping adaptive signals on an independent Acropora dataset. 18
Figure S12. Cross validation of the environmental genomic model. 19
Figure S13. Performance of models explaining Acropora cover decline across Indo-Pacific. 20
Figure S14. Worldwide distribution of Acorpora records from the Ocean Biodiversity Information System (OBIS). 21
Figure S15. Measured and projected maximal annual Degree Heating Week. 22
Figure S16. Temporal projections of heat exposure, frequency of heat-adapted genotypes and genomic vulnerability. 23
Figure S17. Acropora mutations-area relationships (MAR). 24
Figure S18. Acropora z-mar coefficients. 25
Figure S19. Theoretical Allele Frequency Spectrum for a panmictic population. 26
Figure S20. Z-mar coefficient variation by limiting the number of sampling sites. 27
Figure S21. Genetic extinction by area loss simulations. 28
Figure S22. Heat-adapted reefs in conservation and management strategies in 2020. 29

Supplementary Tables

Table S1. Genomic datasets.

Shown are datasets-specific parameters for different processing steps of the study: (A) Dataset characteristics, (B) Filtering thresholds and outputs of reads quality check and mapping, (C) Parameters and outputs of the genotype-by-environment association analysis, (D) output of the mutations area relationship analysis.

Bioproject ID	PRJNA593014	PRJNA702071	PRJNA665778	PRJDB4188	PRJEB37470	PRJNA434194	PRJNA750412
**Authors**	**Fuller et al., 2020**	**Selmoni et al., 2021**	**Drury et al., 2017**	**Shinzato et al., 2015**	**Cooke et al., 2020**	**Matz et al., 2018**	**Torquato et al., 2022**
**A) Datasets characteristics**							
**Species**	*Acropora millepora*	*Acropora millepora*	*Acropora cervicornis*	*Acropora digitifera*	*Acropora tenuis*	*Acropora millepora*	*Acropora downingi*
**# unique coral genotypes**	240	187	179	155	150	85*	61
**# sampling locations**	12	20	22**	12	5	5	10
**Samples per site**	10 to 20	10 to 15	1 to 20	10 to 20	30	20-30	1 to 10
**Region**	Central GBR	New Caledonia	Florida Reef Tract	Ryukyu Archipelago	Central GBR	GBR	Persian Gulf
**Spatial range**	200 km	400 km	400 km	1500 km	500 km	1000 km	800 km
**Sequencing strategy**	WGS (low-coverage)	RAD-Seq (DArT-seq)	RAD-Seq (GBS)	WGS	WGS (low-coverage)	RAD-Seq (2bRAD)	RAD-Seq
**Sequencing machine**	Illumina HiSeq 4000	Illumina HiSeq 2500	Illumina HiSeq 2500	Illumina HiSeq 2000	Illumina HiSeq 2500	Illumina HiSeq 2000	Illumina HiSeq 2000
**B) Filtering and SNP calling**							
**Hard clipping (5' - 3')**	11 - 5	15 - 3	2 - 2	5 - 2	15 - 3	1 - 2	6 - 2
**# of corals after quality filtering**	234	167	128	154	139	85	56
**# of SNPs**	16,338	42,864	9,303	99,810 (23,854,772 before subsampling)	98,285 (1,375,995 before subsampling)	64,947	32,537
**# of coral after outlier filtering**	-	163	76	140	117	79	38
**# sampling sites after filtering**	-	18	14	9	4	5	8
**Pairwise nucleotide diversity (π/bp)**		0.21%	0.45%	0.59%	0.91%	0.14%	0.33%
**C) Genotype-environment association analysis**							
**# of SNPs retained after all. Fr. filtering**	-	9,385	2,773	15,537	15,855	25,597	17,392
**# of colonies retained after allele fr. filtering**	-	156	69	140	116	79	35
**# of latent factors in LFMM**	-	4	4	1	1	1	1
**LFMM genomic inflation factor (λ) before calibration**	-	1.13	1.22	1.19	0.99	1.29	1.39
**LFMM genomic inflation factor (λ) after calibration**		1	1	1	1	1	1
**# of overlapping genomic windows (q<0.1)**	-	39	18	65	56	68	42
**D) Mutations Area Relationship**							
**# of SNPs retained after filtering**	-	40,441	8,474	85,420	68,953	64,430	32,441
**# of colonies retained after filtering**	-	155	63	133	90	79	34
**Raw z-mar**	-	0.23 [0.16-0.28]	0.13 [0.07-0.19]	0.16 [0.10-0.21]	0.06 [0.00-0.11]	0.10 [0.04-0.16]	0.15 [0.09-0.21]
**Panmictic z-mar**	-	0.07 [0.05-0.10]	0.10 [0.07-0.13]	0.05 [0.02-0.07]	0.02 [0.00-0.04]	0.05 [0.03-0.08]	0.08 [0.05-0.11]

* includes samples collected in 2002, excluding 24 samples collected in 2009 and 2014.

** samples collected within 5km of distance were assigned to the same sampling site.

Response variable	PCoA-1			PCoA-2		
	P (Longitude)	***P (Latitude)***	***AIC***	***P (Longitude)***	***P (Latitude)***	***AIC***
**Full model**	2.02E-22	1.26E-05	-1349.13	0.733068	0.024438	-1290.71
**Model without Species**	2.02E-22	1.26E-05	-1351.13	0.960467	0.00847	-1290.09
**Model without Dataset**	1.64E-13	4.65E-01	-1337.97	0.170672	0.126376	-1290.21
**Model with Lon/Lat**	*NA*	*NA*	-1342.43	*NA*	*NA*	-1290.91

Table S2. Coefficients of models explaining genetic distances between samples.

Shown are the coefficients of linear mixed models used to explain the genetic principal coordinate axes (PCoA-1 and PCoA-2) of the six *Acropora* datasets. For every model, shown are the p-value associated with the effects of longitude and latitude, and the overall Akaike Information Criterion (AIC). The full model separates samples based on geography (longitude and latitude), species and dataset. The alternative models remove each one of these factors.

Table S3. Gene ontology enrichment analysis.

Shown are the eleven gene ontology (GO) terms found as enriched in the 85 genomic regions where adaptive signals overlapped from different datasets. For every GO term, the table shows the ID, the description, the number of genes with the annotation across the genome, the test score of the one-sided SetRank enrichment test (setRank), with the corresponding p-value and the FDR adjusted p-value.

Gene Ontology ID	Term description	Size	setRank	P-value	Adj P-value
GO:0016779	nucleotidyltransferase activity	30	0.04718	2.50E-05	0.000274
GO:0031072	heat shock protein binding	38	0.087284	1.31E-04	0.001315
GO:0051082	unfolded protein binding	93	0.04718	1.31E-04	0.001315
GO:0001640	adenylate cyclase inhibiting G protein-coupled glutamate receptor activity	15	0.15962	5.06E-04	0.003539
GO:0099507	ligand-gated ion channel activity involved in regulation of presynaptic membrane potential	10	0.04718	3.93E-04	0.003539
GO:0015277	kainate selective glutamate receptor activity	12	0.060548	4.49E-04	0.003539
GO:0008066	glutamate receptor activity	16	0.086281	5.06E-04	0.003539
GO:0008934	inositol monophosphate 1-phosphatase activity	3	0.087284	7.30E-04	0.005842
GO:0043812	phosphatidylinositol-4-phosphate phosphatase activity	4	0.04718	7.30E-04	0.005842
GO:0004051	arachidonate 5-lipoxygenase activity	3	0.04718	1.35E-03	0.009434

Table S4. Coefficients of *Acropora* cover change models.

Shown are the coefficients of models describing changes in *Acropora* cover in the Indo-Pacific during 2012-2018. For each model shown is the response variable, the fixed effects—with corresponding estimate, standard error, and the two-sided Wald test z-value and p-value—and the random factors. (A) shows the coefficients for a model accounting for heat intensity between surveys, (B) for a model accounting for heat intensity and past heat exposure, (C) for a model accounting for heat intensity and the expected frequency of heat-adaptive genotypes.

A - Max DHW between surveys							
**Response variable**	**Fixed effects**					**Random factors**	
	Coefficient	***Estimate***	***Std. Error***	***z value***	***P-value***	***Factor***	***St. Dev***
Relative *Acropora* cover change between two surveys on different years	(Intercept)	-0.02	0.01	-1.44	0.1489	Reef of survey (intercept)	0.011
	Max DHW between surveys	0	0	-1.18	0.2389	Marine province (intercept)	0.023
						Residuals	0.036

B - Max DHW between surveys x historical maximal DHW							
**Response variable**	**Fixed effects**					**Random factors**	
	Coefficient	***Estimate***	***Std. Error***	***z value***	***P-value***	***Factor***	***St. Dev***
Relative *Acropora* cover change between two surveys on different years	(Intercept)	0.01	0.04	0.21	0.834		
	Max DHW between surveys	-0.01	0	-2.35	0.0187	Reef of survey (intercept)	5e-06
	Max DHW between 1985-first survey	-0.01	0.01	-0.79	0.4288	Marine province (intercept)	0.021
	Interaction: Max DHW between surveys* Max DHW between 1985-first survey	0	0	2.05	0.0407	Residuals	0.036

C - Max DHW between surveys x Expected freq. Heat-adaptive genotypes							
**Response variable**	**Fixed effects**					**Random factors**	
	Coefficient	***Estimate***	***Std. Error***	***z value***	***P-value***	***Factor***	***St. Dev***
Relative *Acropora* cover change between two surveys on different years	(Intercept)	0.12	0.03	4.05	5.17e-05		
	Max DHW between surveys	-0.027	0.01	-4.27	1.95e-05	Reef of survey (intercept)	0.009
	Exp. freq. Adaptive GT	-0.25	0.05	-4.45	8.70e-06	Marine province (intercept)	0.007
	Interaction: Max DHW*Exp. Fr. Ad. GT	0.042	0.01	4.03	5.65e-05	Residuals	0.036

Table S5. Parameters of Mutations-Area Relationship Linear Mixed Model.

Shown are the parameters of the linear mixed models explaining variation in log-transformed mutations (logM) across spatial subsamples from the *Acropora* datasets. In every model (rows), different fixed (logA: log-transformed area of the subsample, logSD: log-transformed mean sequencing depth of samples in the subsample) and random effects were used. The deviance information criterion (DIC) indicates the model's goodness of fit (lower values mean higher fit).

MID	Response variable	Fixed variables	Random effects	DIC
1	logM	-	Dataset	440
2	logM	-	Dataset + Species	440
3	logM	-	Dataset + GenomicSubSample	377
4	logM	-	Dataset + GenomicSubSample + Spatial Index	-2047
5	logM	logA	Dataset + GenomicSubSample + Spatial Index	-3508
6	logM	logA + logSD	Dataset + GenomicSubSample + Spatial Index	-4282

Supplementary Figures

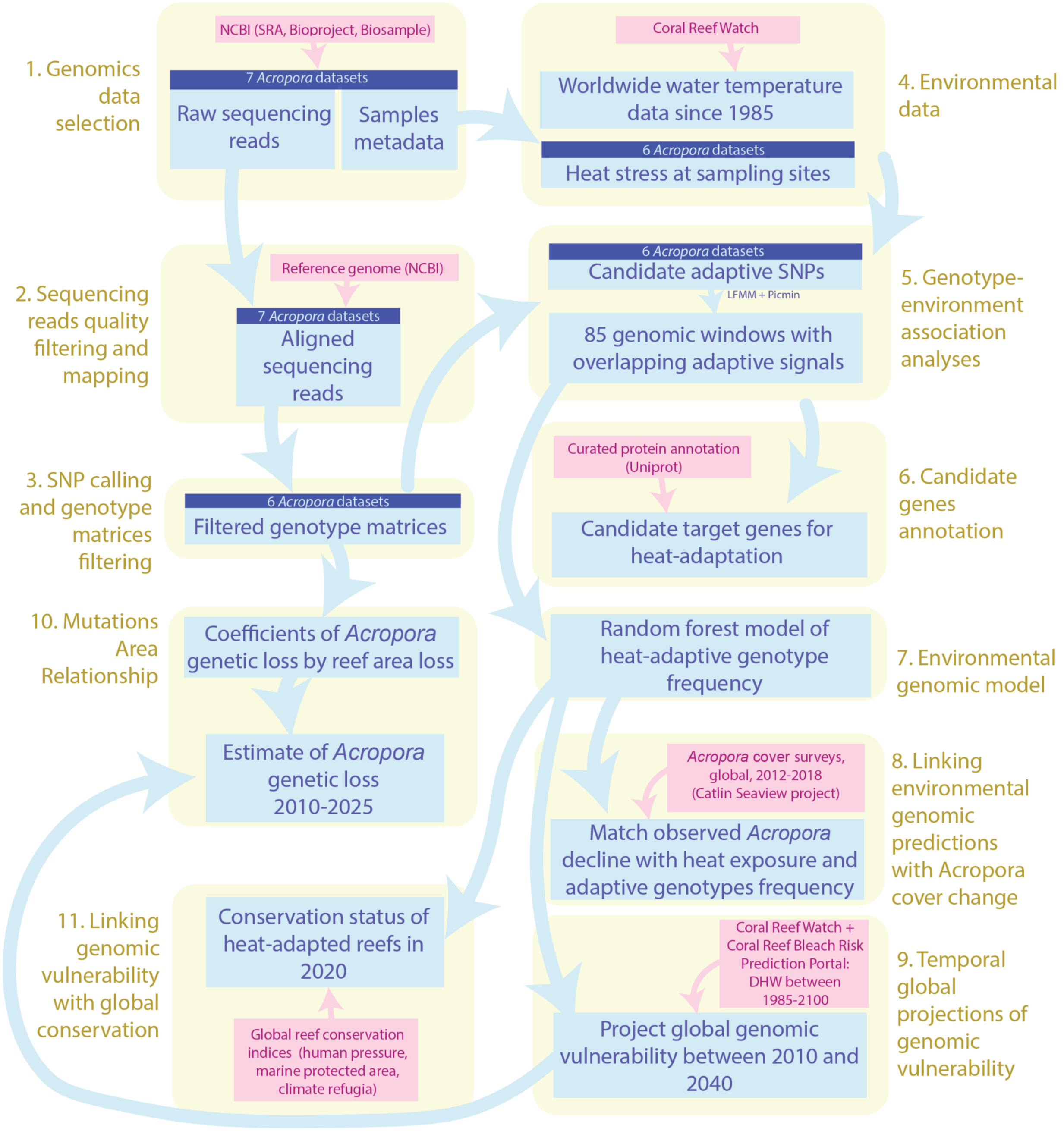

Figure S1. Workflow of the study.

Shown in yellow are the different steps of the study, in red the input from external data sources for the analysis, and in blue the analytical inputs and outputs from the different steps.

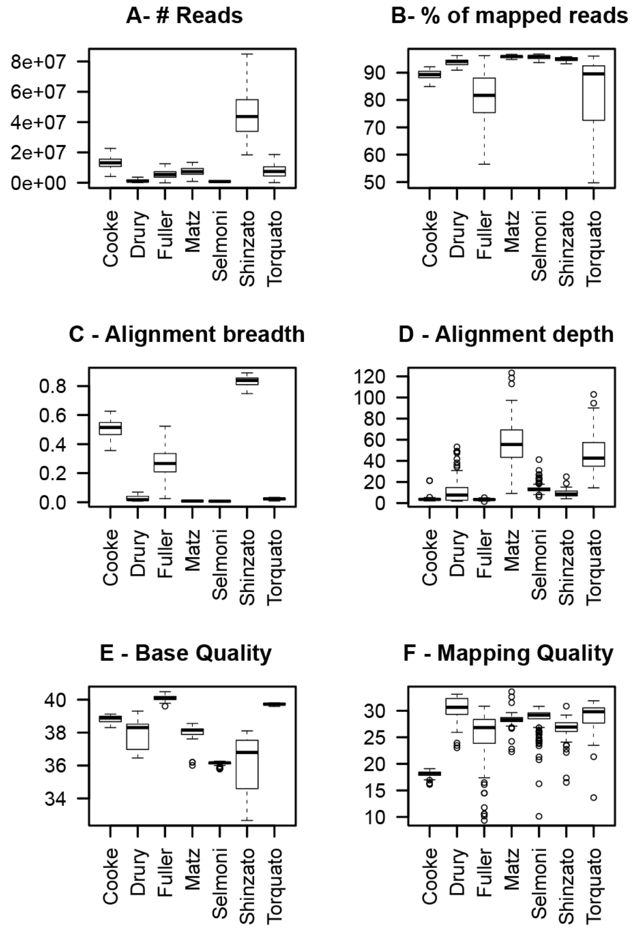

Figure S2. Sequencing read mapping stats.

Shown are the sequencing and alignment statistics by dataset: (A) distribution of number of reads across samples, (B) percentage of reads successfully mapped to the reference across samples, (C) genome breadth covered by alignment across samples, (D) mean sequencing depth across samples, (E) mean base quality scores across samples, (F) mean quality of read-to-reference mapping across samples.

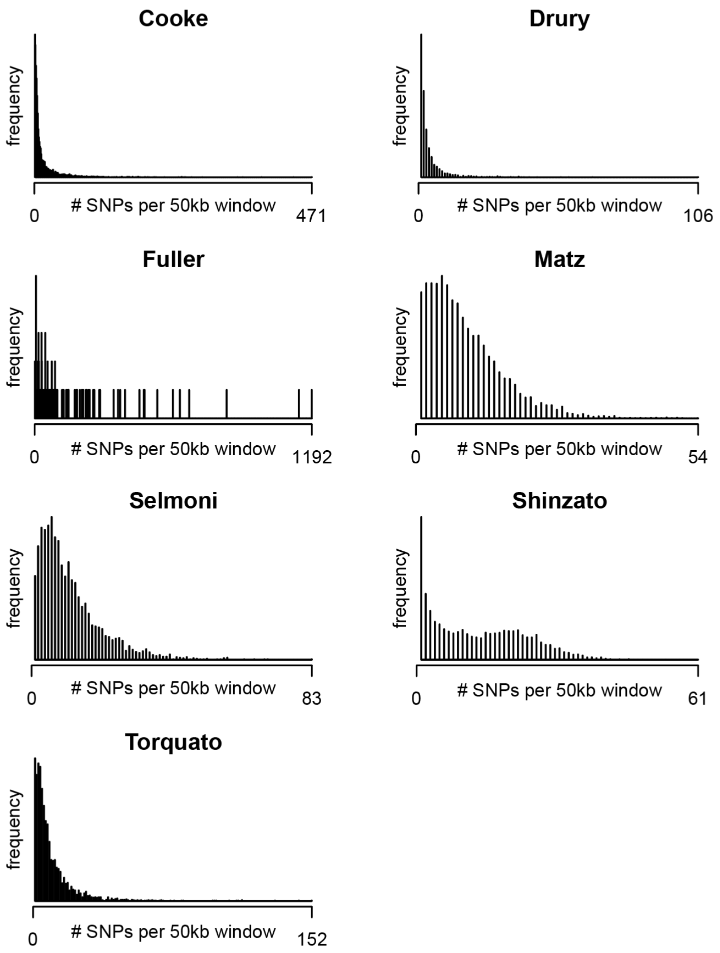

Figure S3. Distribution of SNPs across genomes.

Shown are the number of single nucleotide polymorphisms (SNPs) called across 50 kbs genomic windows in the seven *Acropora* datasets.

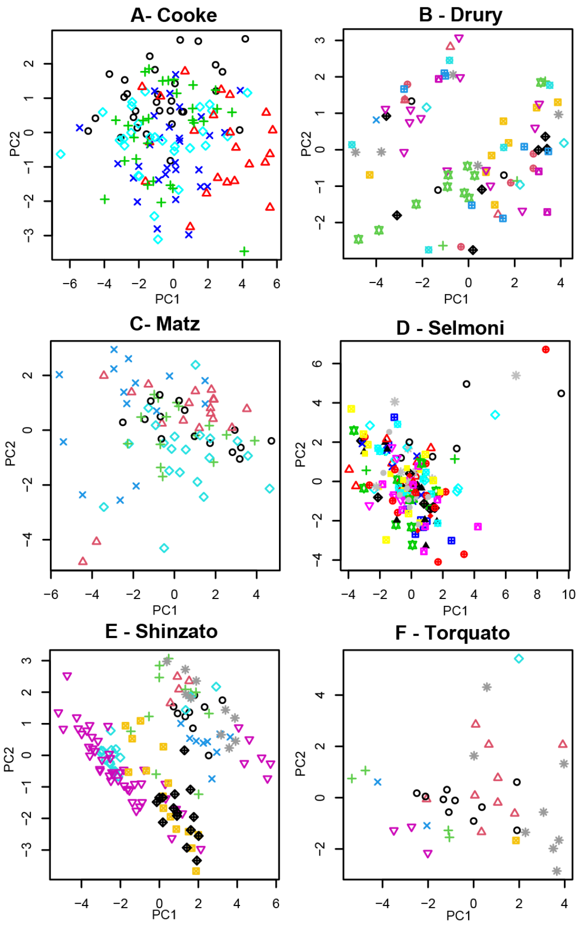

Figure S4. Principal component analysis of sequencing and alignment statistics.

Shown are the main two axes of variation of sequencing and alignment statistics across samples from the six *Acropora* datasets. The axes of variation were computed by running a Principal Component Analysis on by-sample sequencing and alignment statistics: number of reads, percentage of mapped reads, alignment breadth, alignment depth, mean base quality, mean mapping quality. Colored symbols correspond to reefs where samples were collected from.

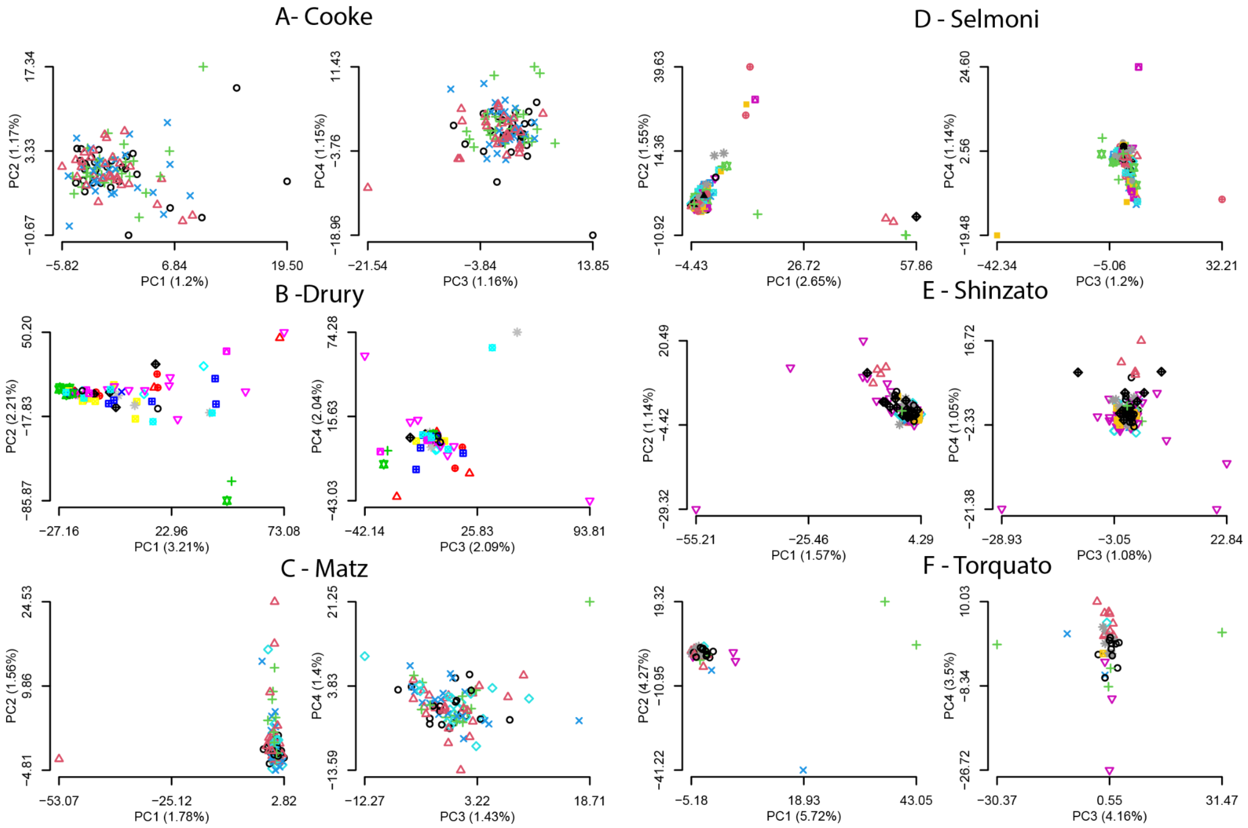

Figure S5. Principal component analysis of genotype matrices.

Shown are the main four axes of genetic variation across samples from the six *Acropora* datasets. The axes of variation were computed by running a Principal Component Analysis on the genotype matrix of every dataset. Colored symbols correspond to distinct sampling locations.

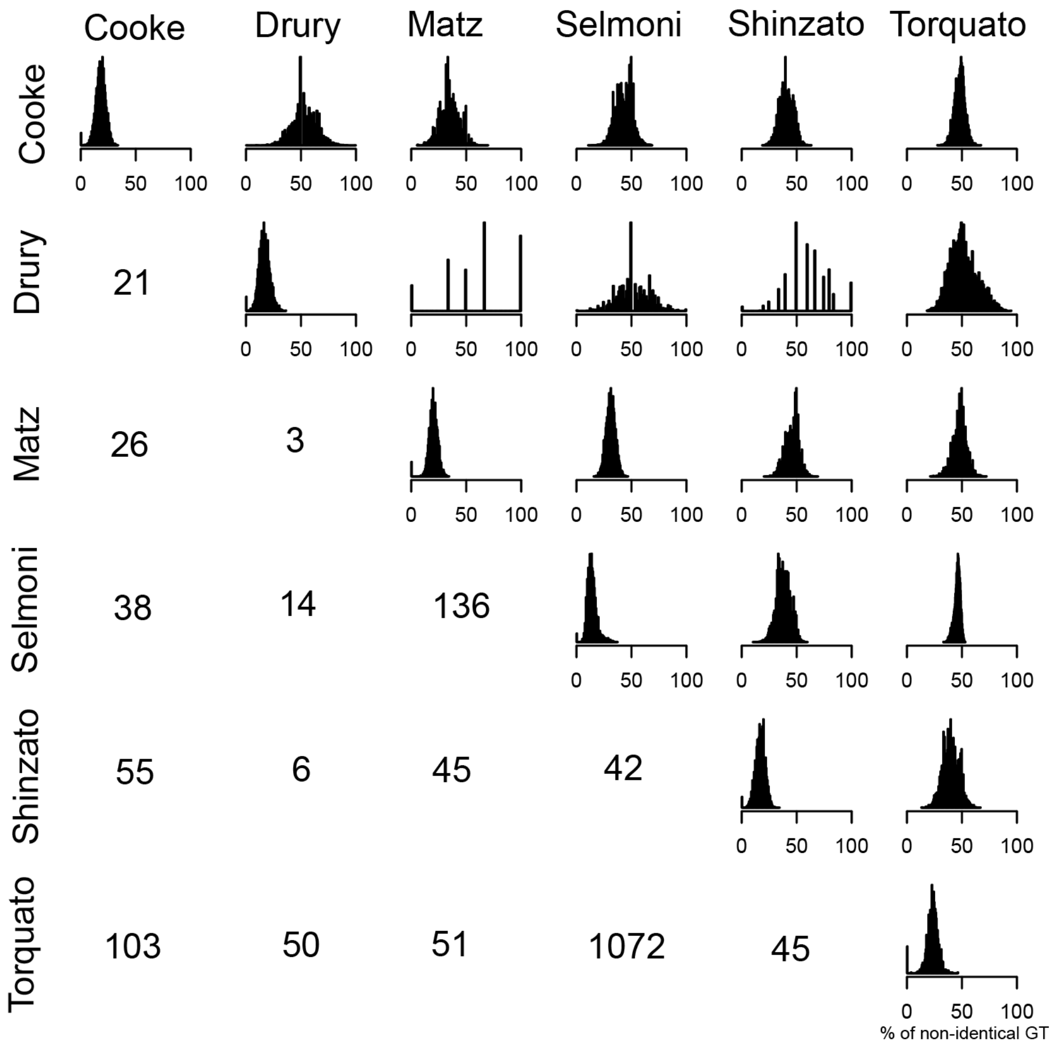

Figure S6. Shared SNPs and % of non-identical genotypes across datasets.

Below the diagonal are shown the number of single nucleotide polymorphisms shared between pairs of datasets. Focusing on these SNPs, the percentage of non-identical genotypes - *i.e.* the genetic distance - was calculated between pairs of samples for different datasets. Above the diagonal are shown the distribution of genetic distance between datasets, the diagonal shows the distribution of genetic distance within datasets.

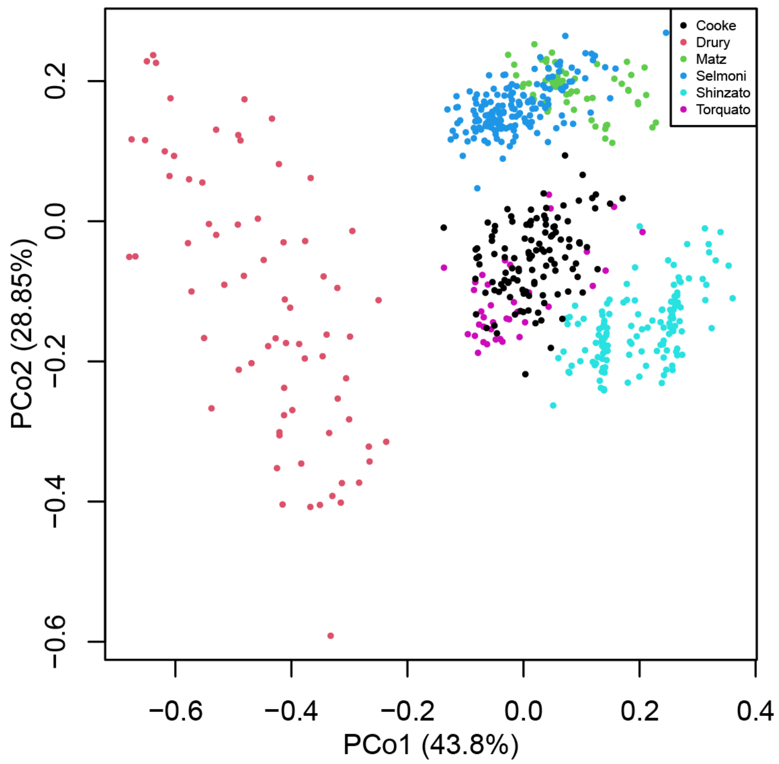

Figure S7. Genetic proximity of all samples from the *Acropora* datasets.

Shown are the main two axes summarizing the genetic distances between the six *Acropora* datasets (colors). The axes were computed by running a Principal Coordinate Analysis on the matrix of genetic distance between all samples, computed as the percentage of non-identical genotypes across the shared single nucleotide polymorphisms.

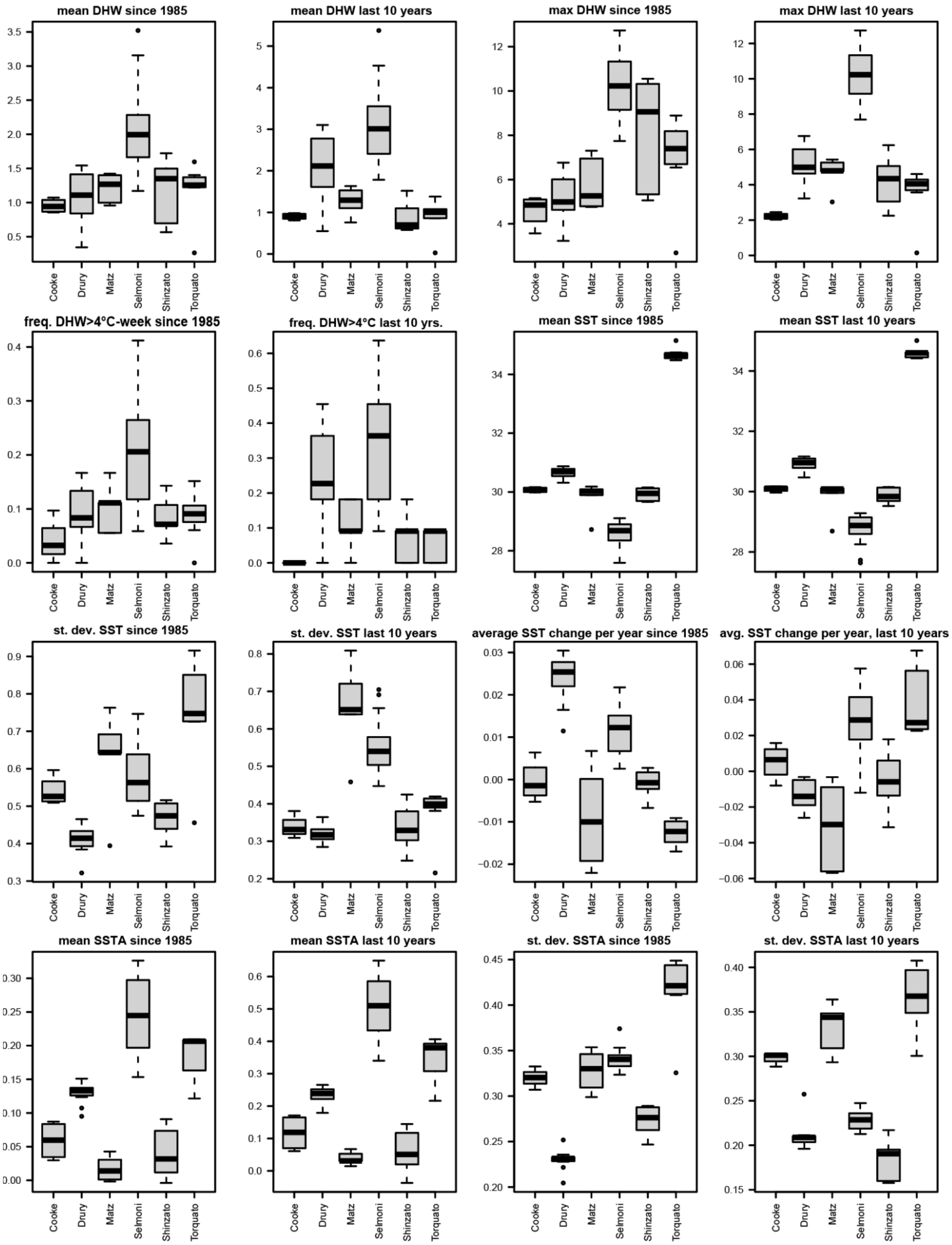

Figure S8. Heat stress variables distribution across datasets.

For the sampling sites of the *Acropora* genomic datasets, shown are the distribution of 16 heat stress variables describing temporal trends of annual maximal Sea Surface Temperature (SST, in °C), annual maximal Degree Heating Week (DHW, in °C-week), and annual mean Sea Surface Temperature Anomaly (SSTA, in °C). Eight variables focus on temporal trends from 1985 until the year of sampling, and eight variables for the ten years before sampling.

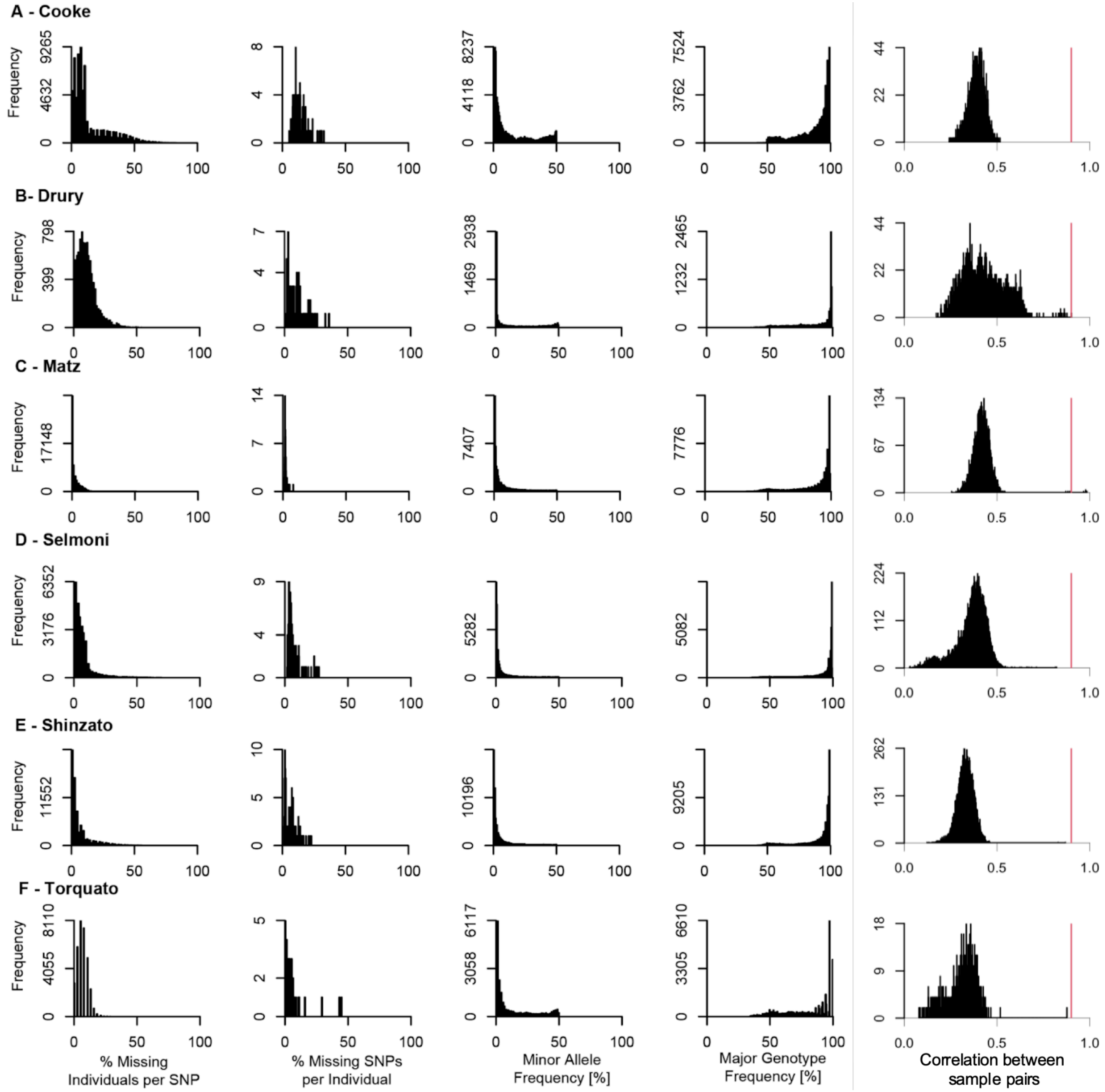

Figure S9. Missing values, allele frequencies statistics and allele frequency correlation

Shown are, for every *Acropora* genomics dataset (A-F), the following statistics of the genotype matrix: percentage of missing individuals per single nucleotide polymorphism (SNP), percentage of missing SNP per individual, minor allele frequency per SNP, major genotype frequency per SNP, and the allele frequency correlation across samples (vertical red line represents the cutoff thresholds for clones R=0.9).

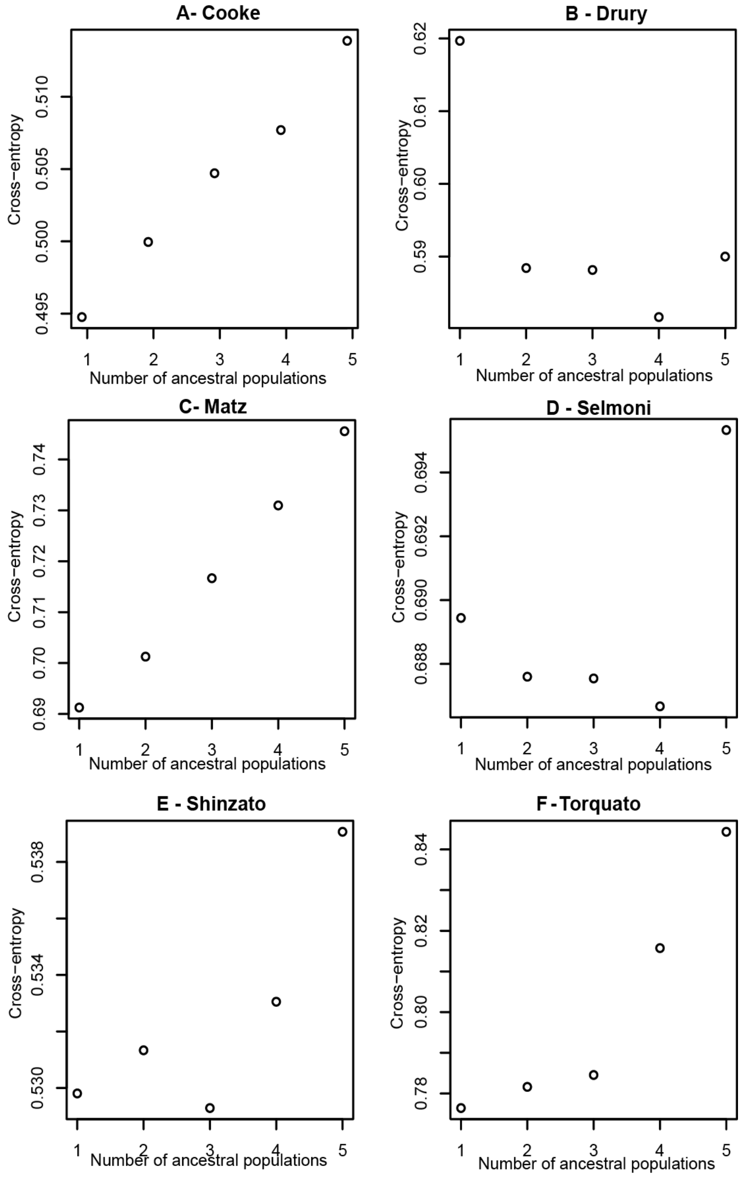

Figure S10. Cross-entropy estimation of the number of ancestral populations.

Shown are the cross-entropy values for different numbers of ancestral populations across the different *Acropora* datasets (A-F). Lower cross-entropy indicates higher goodness of fit of the number of ancestral populations to the genetic diversity observed in the genotype matrix.

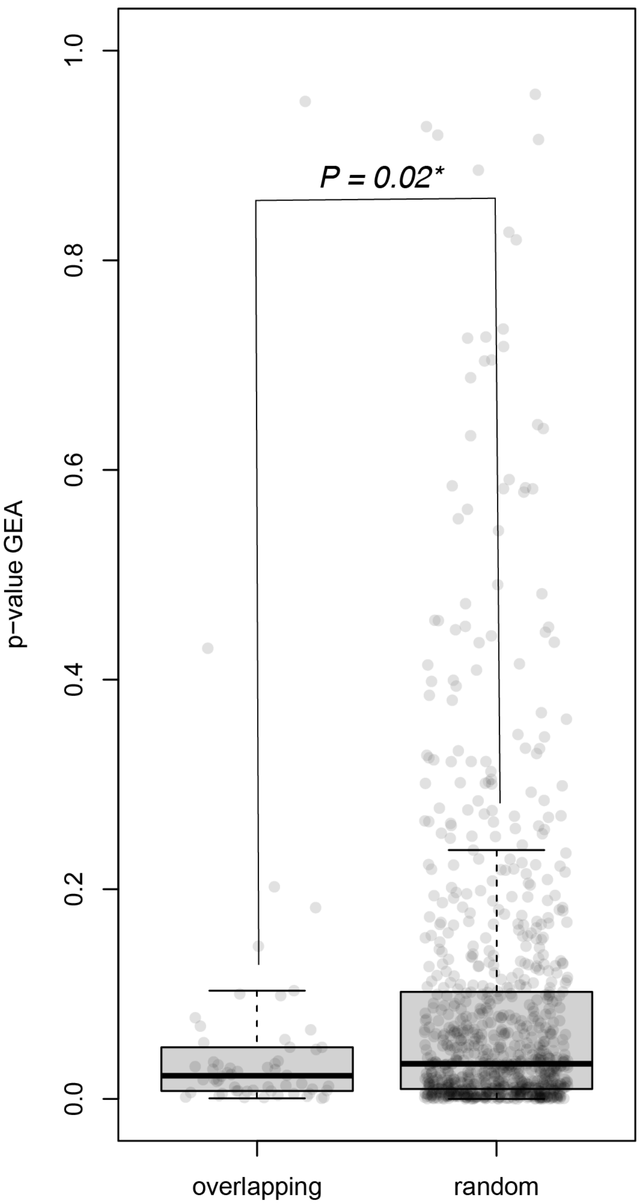

Figure S11. Validation of overlapping adaptive signals on an independent *Acropora* dataset.

Shown are the genome-wide p-values describing the genotype-environment association (GEA) between DHW-max and SNPs from an *A. millepora* population sampled on the central GBR in 2017. Every point corresponds to the lowest GEA p-value found within a 10 kbs genomic window. On the left side: p-value distributions for 85 genomic windows showing significant overlap of heat-adaptive signals in three or more *Acropora* populations from the overlap analysis. On the right side: p-value distribution for 1000 random genomic windows. The distributions of p-values between overlapping and random genomic window were compared using a Wilcoxon rank sum test (test P-value shown on top).

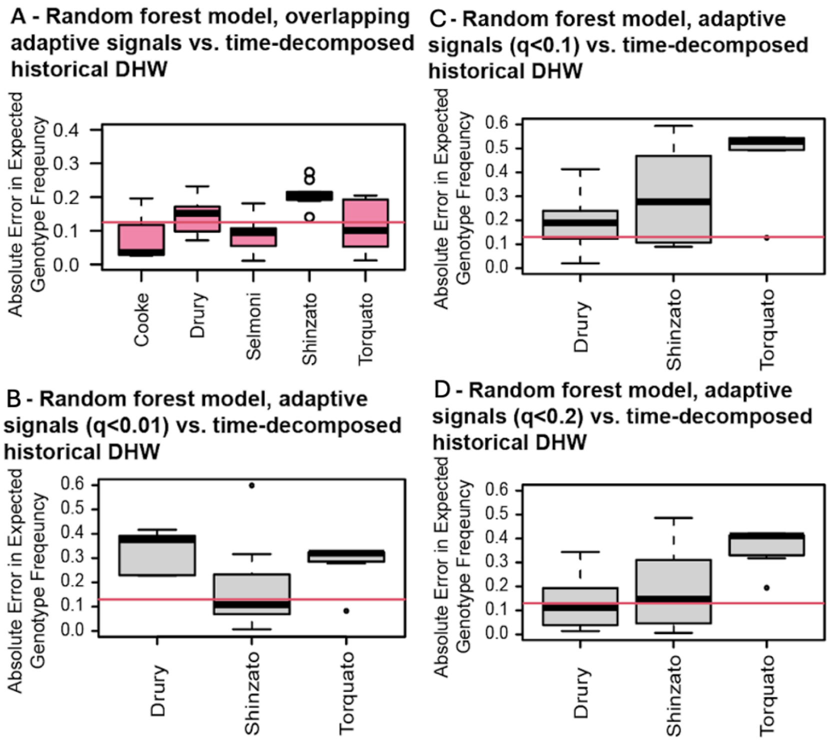

Figure S12. Cross validation of the environmental genomic model.

Shown are the results of the cross validation assessing the predictive power of different environmental genomic models. All models predict the frequency of candidate adaptive genotypes at a given reef from historical Degree Heating Week trends (DHW). For each model, a leave-one-dataset-out cross validation approach was used to calculate absolute errors in predicting the expected genotype frequencies.
In all models, historical maximal DHW was decomposed in 5-year windows (0 to 5, 5 to 10, 10 to 15, 15 to 20 and 20 to 25 years before sampling), and a random forest model estimated genotype frequencies from decomposed DHW trends. In (A), the model is based on candidate adaptive genotypes detected in the same genomic window in at least three datasets (FDR<0.1). In the other panels, models are based on candidate adaptive genotypes detected in any dataset (without overlap analysis) using different FDR thresholds (B = 0.01, C = 0.1, D = 0.2). The red horizontal line is a reference to the mean absolute error in (A).

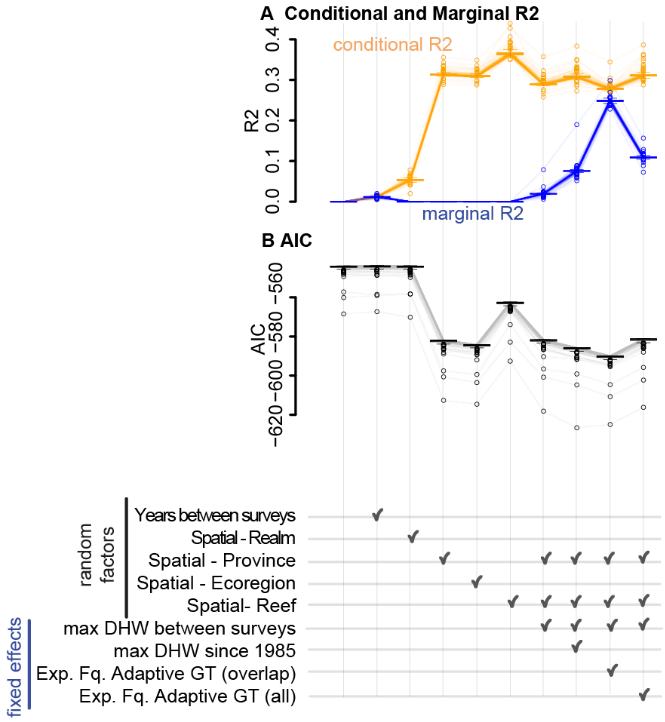

Figure S13. Performance of models explaining *Acropora* cover decline across Indo-Pacific.

(A) shows the distribution of the conditional (orange) and marginal (blue) coefficient of determination (R2) for models using different explanatory variables to describe *Acropora* cover change across the Indo-Pacific. Conditional R2 represents the % of variance explained by the entire model, marginal R2 the % explained by fixed effects. The models use different combinations of explanatory variables: number of years between coral cover surveys, spatial autocorrelation between survey sites at realm, province, ecoregion and reef level, maximal Degree Heating Week (DHW) measured between the dates of the field surveys, the maximal DHW since 1985, and the expected frequency of adaptive genotypes based on an environmental genomic model. The environmental genomic model can be based on candidate adaptive genotypes found in the same genomic regions in different datasets (overlap), or any candidate adaptive genotype found in any dataset (all). The lines on the background represent the conditional R2 estimates obtained using a jackknife resampling approach. (B) shows the distribution of the Akaike Information Criterion (AIC) for the same models.

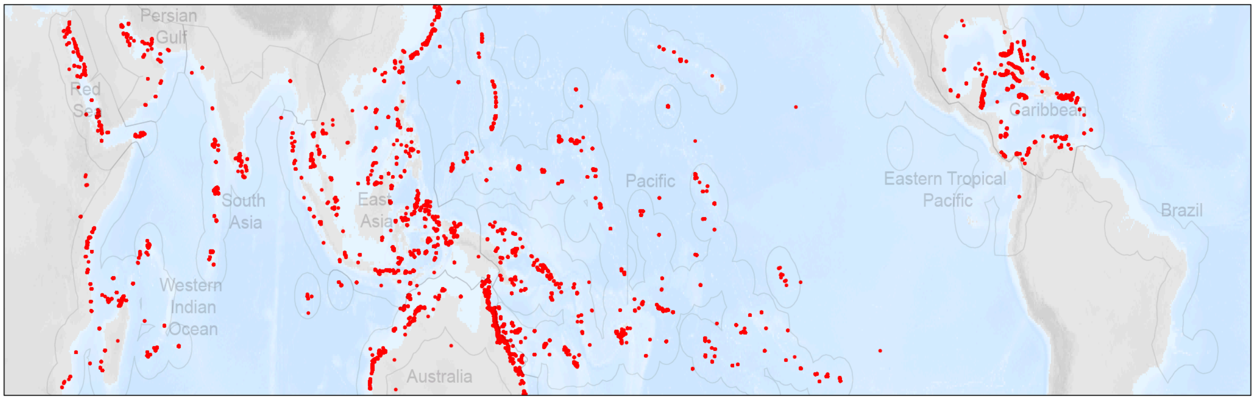

Figure S14. Worldwide distribution of Acorpora records from the Ocean Biodiversity Information System (OBIS).

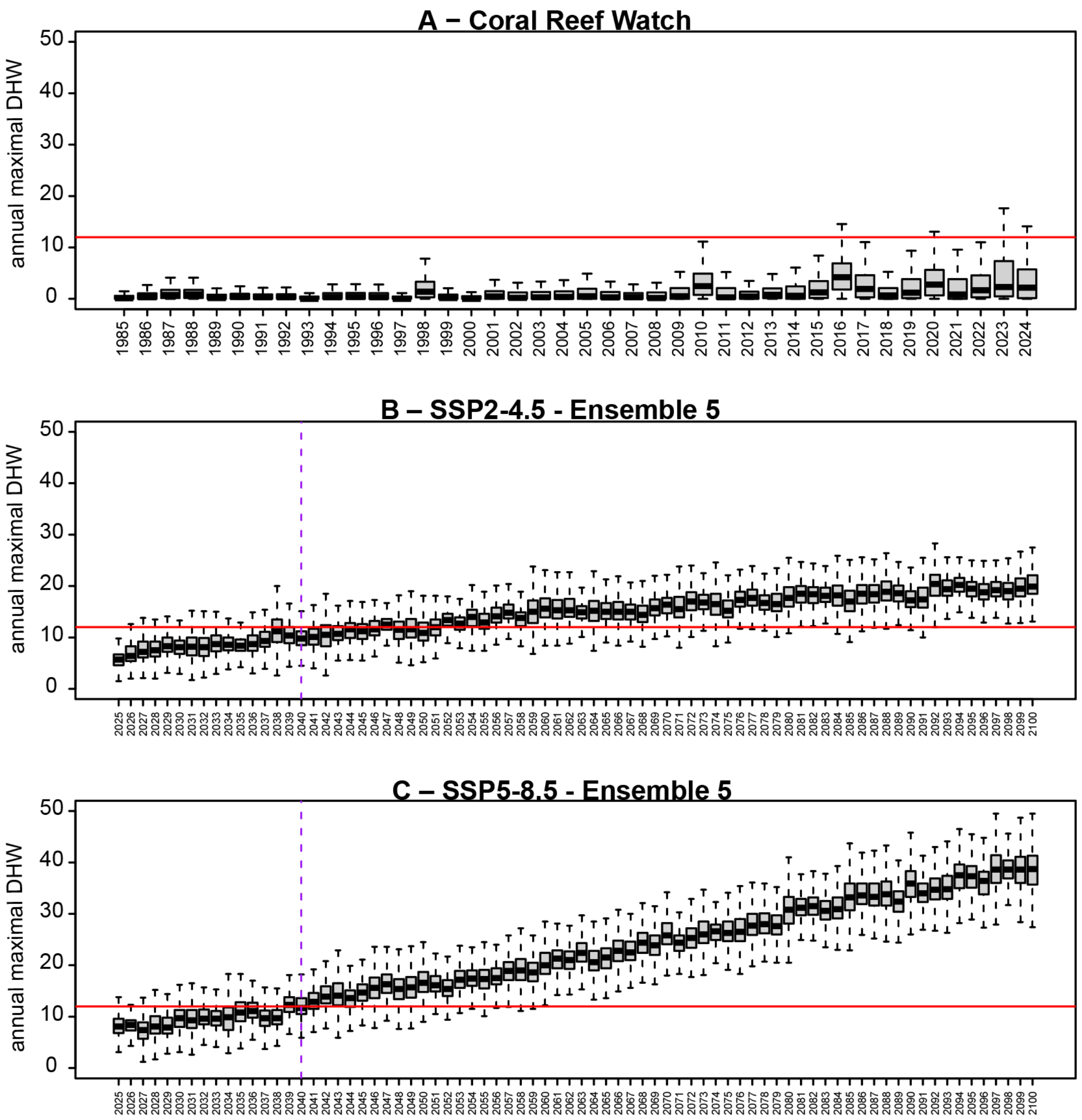

Figure S15. Measured and projected maximal annual Degree Heating Week.

Shown are the year-by-year distribution of measured (A) and projected (B, C) values of maximal Degree Heating Week (DHW) across reefs worldwide. Distributions in (A) are based contemporary measurements (1985-2024) from the Coral Reef Watch, whereas distributions in (B) and (C) from the Coral Reef Bleach Risk Prediction Portal (https://coralbleachrisk.net/), using the Ensemble 5 projections under SSP2-4.5 and SSP5-8.5 scenarios, respectively. The red horizontal line indicates the upper limit of the maximal DHW range used to build the environmental genomic model (~ 12°C-week). After 2040 (purple line), most of the reefs of the world are expected to be exposed to more than 12°C-week per year.

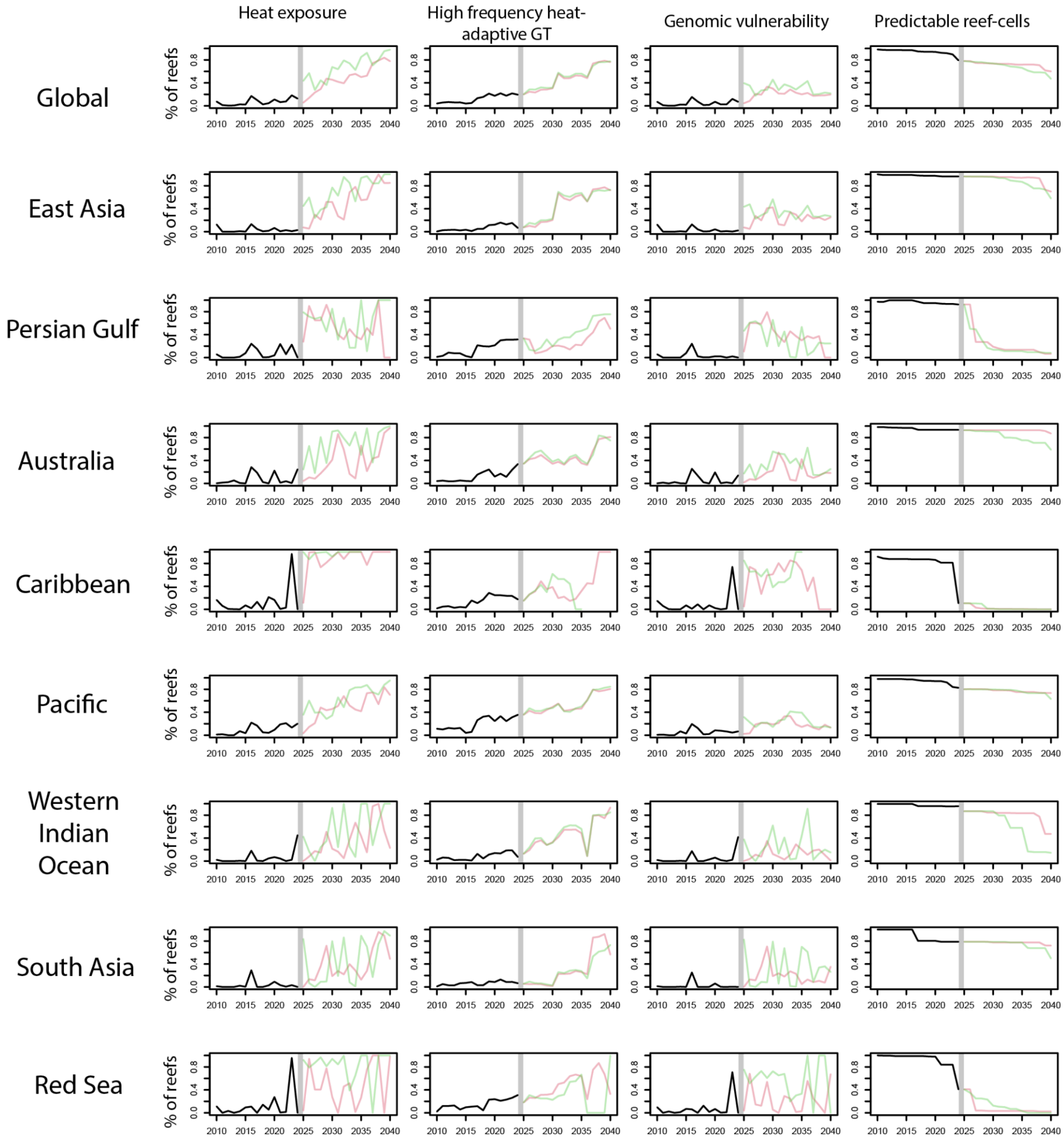

Figure S16. Temporal projections of heat exposure, frequency of heat-adapted genotypes and genomic vulnerability.

Shown are global (first row) and regional (the remaining rows) year-by-year projections between 2010 and 2040. The first column shows the year-by-year fraction of reefs exposed to a severe heatwave (*i.e.*, reefs exposed to a Degree Heating Week-DHW>8°C-week). The second column shows the year-by-year fraction of reefs with a high expected frequency of heat-adaptive genotypes (>70%), based on the environmental genomic model. The third column shows the year-by-year fraction of reefs under genomic vulnerability (*i.e.*, reefs with an expected frequency of heat-adaptive genotype <70%, exposed to DHW>8°C). The fourth column shows the year-by-year fraction of reefs that were usable for calculating the projections (*i.e.*, reefs with thermal history not exceeding the model predictive range). Projections before 2025 are based on the Coral Reef Watch data, projections after 2025 are based on the Coral Reef Bleach Risk Prediction Portal (https://coralbleachrisk.net/), using the Ensemble 5 projections under SSP2-4.5 (red line) and SSP5-8.5 (green line) scenarios.

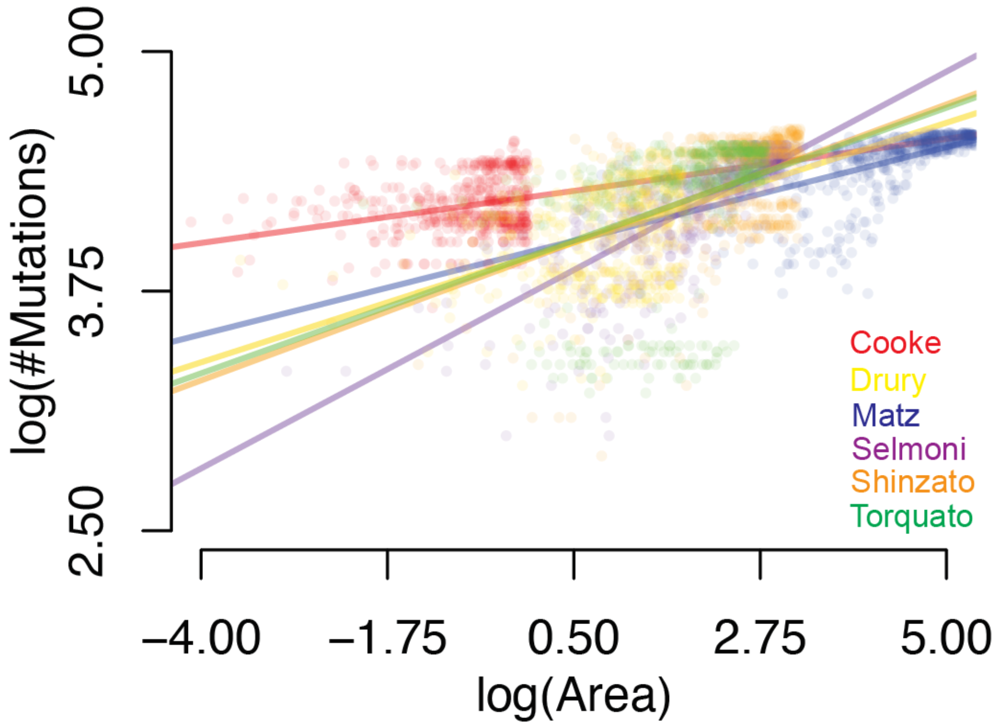

Figure S17. ***Acropora*** **mutations-area relationships (MAR).**

For each dataset (colors), shown are 500 spatial subsamples of the study region with (x-axis) the log-transformed area covered by the spatial subsample, and (y-axis) the log-transformed number of mutations across colonies in the subsampled area. Regression lines correspond to the z-mar parameter of every dataset.

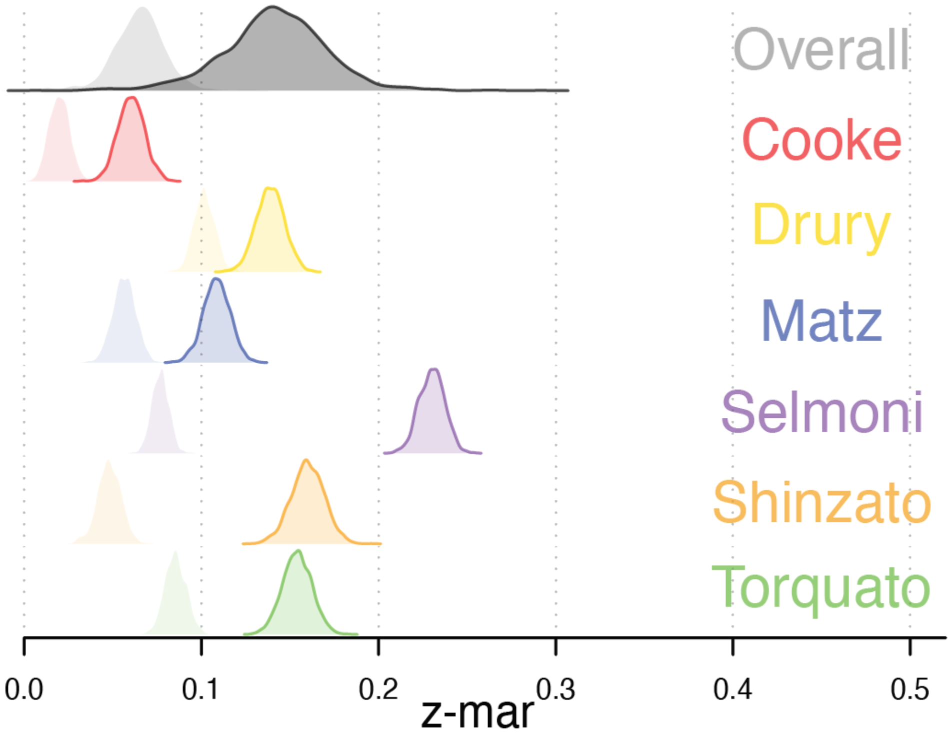

Figure S18. ***Acropora*** **z-mar coefficients.**

Shown are the z-mar coefficients for the six *Acropora* datasets (colored curves), and the overall average *Acropora* z-mar (black curve). The z-mar coefficients were calculated as the regression coefficients of the log-transformed area to the log-transformed number of mutations across datasets. The light-shaded curves represent the z-mar expectations under panmixia.

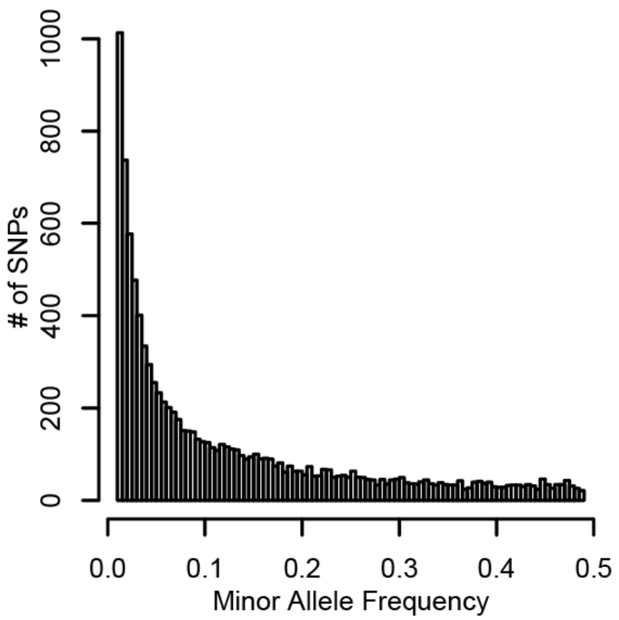

Figure S19. Theoretical Allele Frequency Spectrum for a panmictic population.

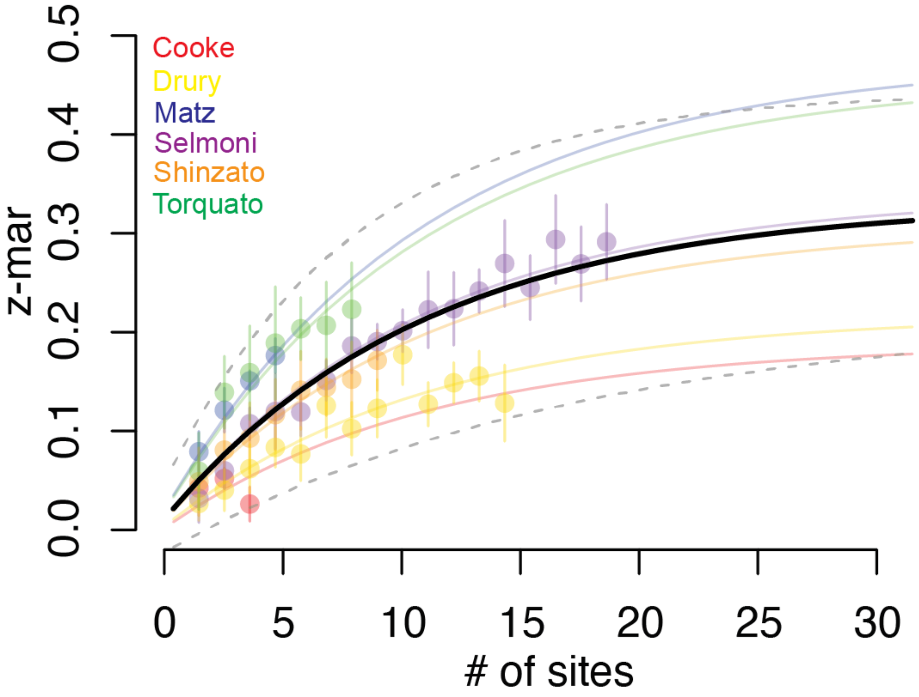

Figure S20. Z-mar coefficient variation by limiting the number of sampling sites.

For every *Acropora* dataset (colors), shown are the z-mar coefficients obtained while limiting the number of sampling sites in the Mutations Area Relationship calculation. The regression lines represent saturation curves of the number of sites-to-z-mar association for every dataset, and the black line represents the overall *Acropora* saturation curve (with intervals of confidence as dotted lines).

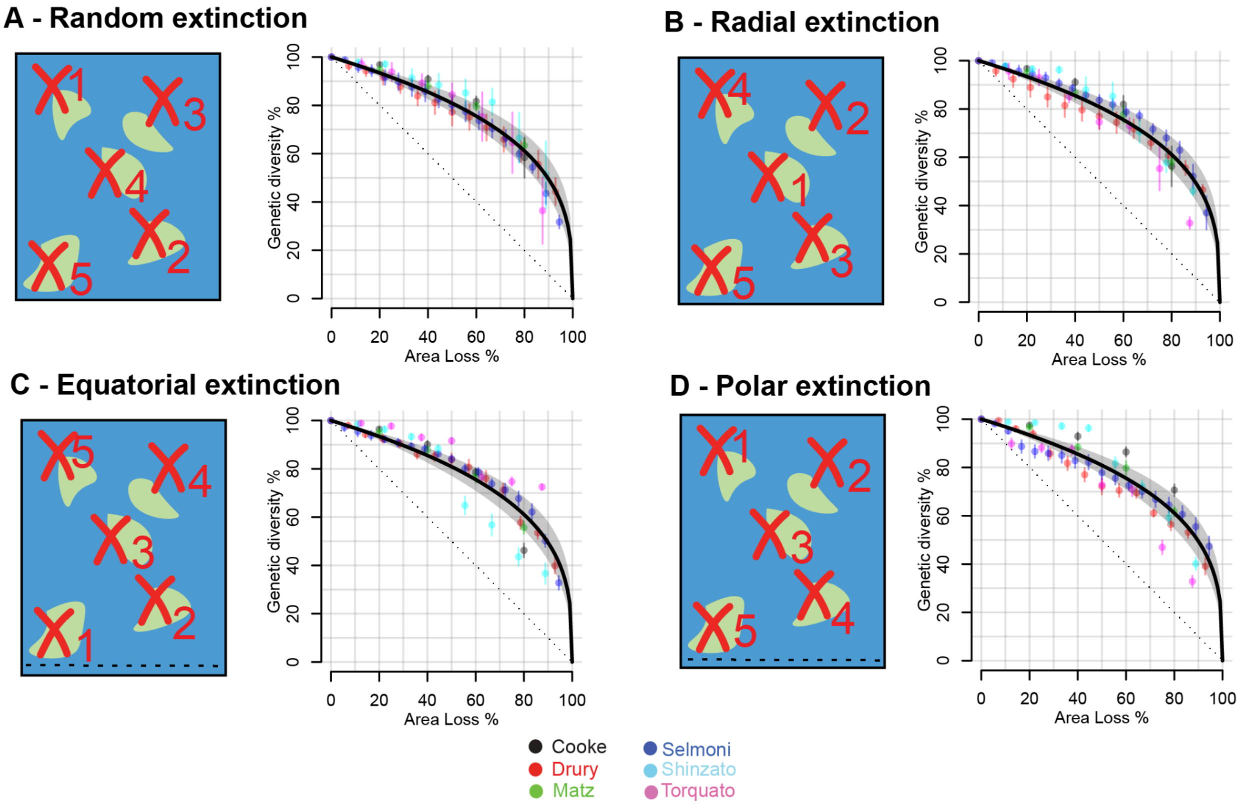

Figure S21. Genetic extinction by area loss simulations.

Shown are the results of stochastic extinction simulations across the *Acropora* datasets (colors in the graphs). In each simulation scenario, the study area of the datasets was progressively reduced, removing individuals from the datasets and counting the amount of mutations (genetic diversity %) lost. The different scenarios show distinct spatial patterns of area loss (red crosses in the drawings display the order of extinctions): (A) reefs are lost following a random order, (B) extinction starts at a given reef, then expands to the neighboring ones; (C) extinction moves from low to high latitudes; (D) extinction moves from high to low latitudes. The black line in the graph represents the expected loss of genetic diversity by area loss according to the average *Acropora* z-mar (with interval of confidence in the shaded area).

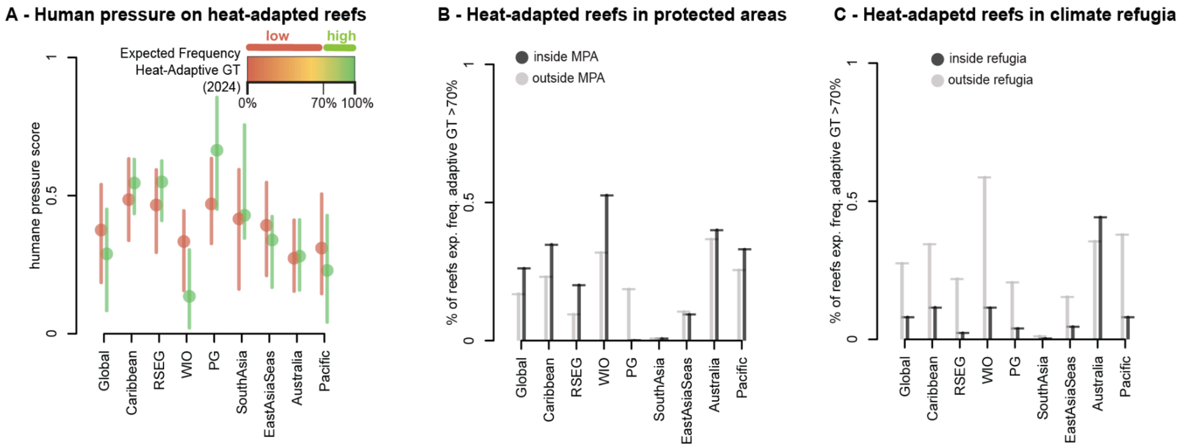

Figure S22. Heat-adapted reefs in conservation and management strategies in 2020.

Shown are three metrics of the conservation status of heat-adapted reefs (*i.e.,* reefs with an expected frequency of heat-adaptive genotypes ≥70%) and non heat-adapted reefs (*i.e.*, expected frequency <70%) across eight oceanic regions. (A) shows the human pressure index (0: low pressure, 1: high pressure) in heat-adapted vs. non heat-adapted reefs. (B) shows the fraction of heat-adapted and non-heat adapted reefs that are inside and outside Marine Protected Areas (MPA). (C) shows the fraction of heat-adapted and non heat-adapted reefs that are included or excluded in a portfolio of 50 climate refugia - *i.e.*, reef areas that are projected to withstand climate change, based on a previous study on connectivity and historical and forecasted climate.
