# Supplementary material for: Global coral genomic vulnerability explains recent reef losses
Source: Nat Commun. 2025 Dec 19;17:896. doi: 10.1038/s41467-025-67616-5 (PMC12830885; doi:10.1038/s41467-025-67616-5)
Supplement: Supplementary file 2 — Description of Additional Supplementary Information [file 41467_2025_67616_MOESM2_ESM.pdf]

Description of Additional Supplementary Files

File Name: Supplementary Data 1
Description: **Overlapping adaptive signals.** Shown is the list of the 85 genomic windows where genotype-environment associations were repeatedly found in different datasets. For every genomic window, the table displays the chromosome or the contig on the *A. millepora* reference genome, the start and the end of the window, q-value of the overlap analysis from PicMin, the datasets were overlapping single nucleotide polymorphisms (SNPs) were detected and the position of the most significant SNP by dataset. The table also displays information on genes located in the genomic window: number of genes (#), gene identifier (ID), gene start and end, and annotated proteins. The table also summarizes the results of the differential gene expression analysis of genes (DGE), where "+" indicates up-regulation of the gene under heat exposure, "-" indicates down-regulation, "ns" a non-significant change, and "?" an unknown change.
